# The Effects of 24 Weeks of Non-Face-to-Face Home Exercise on Body Composition, Physical Fitness, Cardiovascular Function, and Blood Profiles in Pre-Metabolic Syndrome Korean Adults: A Pilot Study

**DOI:** 10.3390/healthcare10101963

**Published:** 2022-10-07

**Authors:** Hee-Soo Ryu, Wi-Young So

**Affiliations:** Sports Medicine Major, College of Humanities and Arts, Korea National University of Transportation, Chungju-si 27469, Korea

**Keywords:** blood lipid profiles, home workout, metabolic syndrome, non-face-to-face, physical fitness

## Abstract

Background: This study assessed Korean residents’ health improvements by reducing chronic disease morbidity through customized health management. After identifying each participant’s strength and health through physical examinations and blood pressure tests, the effects of 24 weeks of online, non-face-to-face home workouts on body composition, physical fitness, cardiovascular function, and blood profiles in Korean adults with pre-metabolic syndrome were determined. Methods: Adults living in Chungcheongbuk-do, aged 19–65, and at the pre-metabolic syndrome stage were recruited at the Jeungpyeong-gun Public Health Center, Chungcheongbuk-do, Korea. For 24 weeks, from February 2022 to July 2022, they exercised for 60–70 min, three times a week, on average, at a maximum heart rate of 60–70%. The “Mobile Healthcare” application was used to record exercise time, number of exercises, number of sets, and Rating of Perceived Exertion at the end of each exercise. Body composition, physical fitness, cardiovascular function, and blood profiles were measured before and after participation. Results: There were no significant differences in weight, body mass index, body fat, waist-to-hip ratio, waist circumference, systolic blood pressure, diastolic blood pressure, resting heart rate levels, or glucose levels after participation in the workout (*p* > 0.05). However, muscle strength/handgrip strength, sitting-rising test results, single-leg balance, triglycerides, high-density lipoprotein, low-density lipoprotein, and total cholesterol showed a statistically significant difference (*p* < 0.05). Conclusions: Although the 24 weeks of non-face-to-face home workouts did not positively affect body composition or cardiovascular function, it improved physical fitness and dyslipidemia.

## 1. Introduction

Today, our focus is not just on living a long life, but also on living a mentally and physically healthy and happy one [1]. Although advances in science and technology have extended our lifespans, the emergence and prevalence of various pathogens have also increased. Additionally, metabolic syndrome has become prevalent; it is based on multiple biochemical and physiological characteristics, such as obesity, and is associated with cardiovascular disease and type 2 diabetes. According to the World Health Organization, more than 1.9 billion people worldwide over 18 years of age are overweight, and more than 650 million are obese. As the population is expected to increase, this will also influence the prevalence of secondary diseases caused by obesity, such as hypertension, diabetes, and dyslipidemia. This increases metabolic syndrome [2]. Korea is no exception to such trends. According to the National Health and Nutrition Examination Survey, the prevalence of metabolic syndrome among Korean adults from 2016 to 2018 was 23% for those aged 19 years and older, 27.7% for those aged 30 years and older, and 45.3% for those aged 65 years and older [3]. Currently, many studies are being conducted on various diet and exercise regimens to lower the prevalence of metabolic syndrome [4,5]. These have shown that the most effective method is to combine diet and exercise therapy [6]. Moreover, exercise alone has been verified to positively affect blood pressure, blood sugar, and lipid levels [7,8,9,10].

However, with the onset of the COVID-19 pandemic, the participation rate in physical activity has decreased significantly [11]. In the early days of the epidemic, it was thought that obese people with underlying conditions such as diabetes, high blood pressure, and cerebrovascular disease were at increased risk of becoming seriously ill if they became infected with the virus. However, recent research results suggest that obesity itself can be dangerous to those infected with COVID-19, even without an underlying disease [11,12]. Most agree with the claim that non-face-to-face healthcare using smart devices will be at the epicenter of a new, normal trend in the post-COVID-19 era, based on the prediction that a complete end to the virus may be difficult [13]. Therefore, differentiated exercise programs that consider the individual’s physical condition will be an ongoing necessity.

As social distancing has become commonplace, various social platforms have increased the information available on exercise and health to promote better health; however, it is difficult to define one’s condition at these sites using only generalized information. For this pilot study, a customized healthcare service program using information and communications technology (ICT) was developed and applied after identifying the physical fitness and health of the participants through physical examinations, blood profiling, and blood pressure tests. This program promptly identifies the exercise progress of participants and provides feedback to help participants understand their current exercise progress and motivate them to improve their health and physical fitness, thereby inducing continuous exercise participation. The program aims to improve the health of local Koreans and reduce the morbidity related to chronic diseases, ultimately contributing to the health and economic well-being of the country.

## 2. Materials and Methods

### 2.1. Participants

The study was conducted on adults living in Chungcheongbuk-do, aged between 19 and 65 and at a pre-metabolic syndrome stage with at least one health risk factor, from February 2022 to July 2022. Our study established the parameters of hypertension, diabetes, obesity, and dyslipidemia as the criteria for diagnosing metabolic syndrome, as presented in the Third Report from the National Cholesterol Education Program Expert Panel on the Detection, Evaluation, and Treatment of Hyperglycemic Cholesterol in Adults [14,15]. The research was designed as a “pre- and post-single group” study. To determine the appropriate number of participants needed, we set our power at 0.80, effect size at 0.40, considered two tails, and set a significance of 0.05 using the G∙POWER program (G∙POWER program 3.1.9.7, Heinrich Heine University, Düsseldorf, Germany). We needed at least 52 study participants [16]; therefore, we conducted the program experiment with 58 participants. The study protocol was approved by the Ethics Committee of the Korea National University of Transportation (approval number: KNUT IRB 2022-01) and conformed to the standards set by the latest revision of the Declaration of Helsinki. All participants in the study were informed of its purpose and content, and those who voluntarily participated were asked for consent before proceeding with the program in the Jeungpyeong-gun Public Health Center, Chungcheongbuk-do, Korea.

The inclusion criteria were as follows: aged ≥20 or ≤65 years and those with one or more of the considered risk factors without hypertension or diabetes (systolic blood pressure (SBP), ≥130 mmHg; diastolic blood pressure (DBP), ≥85 mmHg; fasting blood glucose levels, ≥100 mg/dL; waist circumference, male ≥90 cm, female ≥85 cm; triglyceride levels, ≥150 mg/dL; and high-density lipoprotein cholesterol (HDL-C) levels, male <40 mg/dL, female <50 mg/dL). The exclusion criteria were as follows: aged <20 or >65 years and taking ongoing hypertension or diabetes medications.

### 2.2. Measurements

#### 2.2.1. Anthropometric Characteristics

Among the physical characteristics, height was measured using an ultrasonic extensometer (BSM-230, BioSpace, Seoul, Korea), and other physical characteristics and body composition variables were analyzed using direct segmental multi-frequency biological impedance analysis (InBody 770, BioSpace, Seoul, Korea). All metal and accessories worn by the participants were removed. In addition, exercise was prohibited for 12 h before the measurements, and water intake was prohibited 4 h before [17].

#### 2.2.2. Cardiovascular Function

SBP and DBP were measured using an automatic sphygmomanometer (Boso Carat Professional E, BOSCH + SOHN GmbH Co. KG, Bahnhofstraße, Germany) before and after participation in the program. After raising the sphygmomanometer to the heart’s height, the participants rested for 5–10 min without talking or moving, and the brachial artery blood pressure was measured.

### 2.3. Physical Fitness

#### 2.3.1. Handgrip Strength

The maximal contractility test is a useful predictive tool for screening vital signs in middle-aged and older people [18,19]. This study used a grip strength dynamometer (T.K.K–5401, Takei, Japan) as a grip force measuring instrument. After setting the dynamometer to 0, the second joint of the right finger grips the handle. The elbows are positioned straight at a 15° angle from the body. Thereafter, the participant holds the dynamometer for a maximum of 5 s, while avoiding Valsalva maneuvers at this point. The value is measured twice in total, and the higher value is recorded.

#### 2.3.2. Sitting-Rising Test (SRT)

The SRT is a simple and safe tool developed to simultaneously evaluate non-aerobic exercise components, such as strength, power, flexibility, and balance, with only one test [20,21]. Its efficacy and validity have already been proven; the SRT is a reliable and sensitive method for evaluating the quality of sitting and rising motions [22]. The participants stand barefoot on the mat, cross their legs without holding onto anything, sit with their buttocks touching the mat, and then stand up again to the original position. In this case, the participant does not have to be concerned about movement speed. All participants start with 10 points, with 1 point deducted when the legs, hands, forearms, knees, or sides are used or if the hands are placed on their knees or thighs. When balance was lost, 0.5 points were deducted. A score of 8–10 was recorded as good, 3.5–7.5 as fair, and 0–3 as poor.

#### 2.3.3. Single-Leg Balance (Closed Eyes)

Single-leg balance is a representative method for measuring balance [23]. A decrease in balance tends to weaken an older person’s ability to maintain posture and body balance, limits daily life activities, and increases the risk of a fall [24]. Regular exercise helps improve cardiorespiratory function, increase muscle strength in the upper and lower body, and improve balance by developing proprioception. As mentioned earlier, balance measurement and evaluation are essential because they are directly related to the risk of falls related to age [25,26]. During the test, the participants stood barefoot on a mat near the wall to prevent falls, and stood on their left foot, bending their right leg to 90°; they closed their eyes and held the position for as long as possible. The test was conducted twice, with the highest value recorded. During the test, the researcher assisted the participants to prevent accidents.

#### 2.3.4. Blood Profiles

Participants were instructed to fast for at least 9 h before the blood tests. After resting for 10–20 min before blood collection, 0.5 µL of blood was drawn from the tip of the ring finger using lancets. Subsequently, the glucose, triglyceride (TG), high-density lipoprotein (HDL), low-density lipoprotein (LDL), and total cholesterol (TC) levels were determined using a blood glucose meter (STANDARD GlucoNavii NFC, Seoul, Korea).

#### 2.3.5. Contact-Free Home Workout

With growing interest in health, social platforms are already full of information about exercise. However, the most important factor when exercising is to set the appropriate frequency and intensity according to your body condition for avoiding injury. In this study, screening tests and counseling were conducted in advance to prevent the risk of injury. For our research, we developed an exercise program to ensure the most benefits based on body weight for safe and easy workouts at home. In addition, for the researcher to monitor the participant’s exercise participation, the participant was instructed to record exercise time, number of exercises, number of exercise sets, and Rating of Perceived Exertion (RPE) value at the end of each exercise using the application “Mobile Healthcare.” The data were immediately transmitted to the researcher; the participants were consulted with on managing and supervising their non-face-to-face exercise, intensity control, and exercise advice.

#### 2.3.6. Exercise Program

To set individual exercise intensity, we derived the target heart rate using the maximum heart rate formula according to age (HRmax = 220 − age) and the Karvonen formula ([220 − age − resting heart rate] × [exercise intensity] + resting heart rate) [27,28]. According to the recommendations of the American College of Sports Medicine, the intensity of exercise was selected as 60–70%, which is the most effective for weight loss, is associated with a low risk of injury, and is relatively easy to supervise [29]. Subsequently, as physical fitness increased, the exercise load was increased at 8-week intervals to maintain the exercise intensity of 60–70%, and four sets of 15–20 repetitions maximum were performed. The main strength exercise equipment used was 2–3 kg dumbbells and a Theraband. In Table 1, the exercise program includes exercises related to the upper extremities, such as abdominal muscles (crunch, lying leg raise, standing side bend), arms (bicep curl, dips, standing one-arm triceps extension, triceps extension), chest (push-up), and shoulders (bent-over lateral raises, front raises, lateral raises, seated shoulder presses, shoulder presses). For the main strength exercises related to the lower extremities, the muscles used consisted of the gastrocnemius and soleus muscles (calf lift), hamstrings (bridge, good morning, lying leg cycle), and quadriceps (ball squat, chair squat, deadlift, lunge, squat).

### 2.4. Statistical Analysis

Statistical analyses were conducted using SPSS version 23.0 (IBM Corp., Armonk, NY, USA). The means and standard deviations for all variables were expressed and calculated using an independent or paired *t*-test to compare pre-training and 24 weeks post-training. Moreover, this study showed stratification by sex to induce participation in continuous exercise for improving health and to take advantage of customization in the digital age. The statistical significance was set at *p* = 0.05.

## 3. Results

The physical characteristics of the participants are summarized in Table 2. The results of the measured anthropometric characteristics after the non-face-to-face exercise training program (aerobic exercise and strength exercise) are presented in Table 3. In terms of weight, the pre-program mean value was 67.12 ± 13.23 kg, and the post-program value was 66.44 ± 12.51 kg, showing no statistically significant difference (*p* = 0.138). In terms of body mass index, the pre-program mean value was 25.59 ± 3.65 kg/m², whereas the post-program value was 25.46 ± 3.59 kg/m², showing no statistically significant difference (*p* = 0.176). In terms of body fat, the pre-program mean value was 33.23 ± 7.39%, whereas the post-program was 32.77 ± 7.87%, showing no statistically significant difference (*p* = 0.281). In terms of waist-hip ratio, the pre-program mean value was 0.91 ± 0.06, and the post-program was 0.91 ± 0.06, showing no statistically significant difference (*p* = 0.393). For the waist, the pre-program mean value was 88.17 ± 11.03 cm, and the post-program was 88.11 ± 11.09 cm, showing no statistically significant difference (*p* = 0.921).

Table 4 presents the changes in the cardiovascular function variables after 24 weeks of non-face-to-face exercise. Regarding SBP, the pre-program mean value was 122.93 ± 10.73 mmHg, and the post-program value was 121.41 ± 9.12 mmHg, showing no statistically significant difference (*p* = 0.218). Regarding DBP, the pre-program mean value was 81.21 ± 7.67 mmHg, whereas the post-program value was 81.09 ± 7.93 mmHg, showing no statistically significant difference (*p* = 0.874). For the resting heart rate, the pre-program mean value was 75.43 ± 9.83 bpm and post-program value was 74.28 ± 10.37 bpm, showing no statistically significant difference (*p* = 0.383).

Table 5 presents the changes in physical fitness variables after 24 weeks of non-face-to-face exercise. In terms of muscle strength (handgrip), the pre-program mean value was 29.56 ± 9.54 kg, and the post-program value was 30.38 ± 9.48 kg, indicating an improvement of about 1 kg and a statistically significant difference (*p* = 0.002). For the SRT, the pre-program mean value was 7.81 ± 1.71 points, whereas the post-program value was 9.05 ± 1.13 points, indicating an improvement of approximately 2 points and a statistically significant difference (*p* < 0.001). For single-leg balance, the pre-program mean value was 12.39 ± 9.91 s, whereas the post-program value was 14.72 ± 11.82 s, indicating an improvement of approximately 2 s or more, showing a statistically significant difference (*p* = 0.035).

Table 6 presents the changes in the blood profile variables after 24 weeks of non-face-to-face exercise. In terms of glucose, the pre-program mean value was 97.16 ± 7.81 kg, and the post-program value was 98.02 ± 10.25 mg/dL, showing no statistically significant difference (*p* = 0.528). Regarding TGs, the pre-program mean value was 99.62 ± 48.01 mg/dL, and the post-program mean value was 74.36 ± 48.56 mg/dL, indicating a decrease of about 25 mg/dL or more and showing a statistically significant difference (*p* < 0.001). Regarding HDL, the pre-program mean value was 53.91 ± 16.98 mg/dL, whereas the post-program mean value was 49.14 ± 16.94 mg/dL, indicating a decrease of approximately 4 mg/dL and showing a statistically significant difference (*p* = 0.002). In terms of LDL, the pre-program mean value was 113.20 ± 34.14 mg/dL and post-program mean value was 102.29 ± 37.28 mg/dL, indicating a decrease of about 11 mg/dL and showing a statistically significant difference (*p* = 0.043). For TC, the pre-program mean value was 187.60 ± 33.44 mg/dL, whereas the post-program mean value was 167.14 ± 38.34 mg/dL, indicating a decrease of about 20 mg/dL, which is a statistically significant difference (*p* < 0.001).

## 4. Discussion

Some studies have shown that anthropometric characteristics and cardiovascular functions reflect positive changes after an exercise program is implemented for a certain period [7,30]. Our study investigated the effect of 24 weeks of non-face-to-face home exercise on body composition, physical fitness, cardiovascular function, and blood profiles among Korean adults with pre-metabolic syndrome. Although the anthropometric characteristics and cardiovascular functions improved, there were no statistically significant differences pre- and post-program. One of the reasons for this may be that the participants had other underlying diseases that limited the effects of exercise, such as high blood pressure, diabetes, dyslipidemia, and obesity. For example, in participants who had undergone kidney transplants, small arterial stiffness improved significantly due to exercise, but blood pressure, lipid profiles, blood glucose levels, kidney function, body weight, and body mass index did not improve significantly [31]. Second, a change in diet was not part of the program. It is well known that drinking reduces liver lipid oxidation and interferes with fat and carbohydrate metabolism, and habitual drinking exceeding energy requirements increases lipid storage, weight gain, and the incidence of hypertension [32,33].

Moreover, smoking is a potent risk factor for atherosclerosis, which causes arteriosclerosis, endothelial dysfunction, and inflammation, and induces hypertension by stimulating the sympathetic nervous system [34,35]. In our study, we did not screen out participants with diseases other than the risk factors for metabolic syndrome, nor did we impose drinking, smoking, or dietary restrictions. Thus, these factors may have contributed to our results’ lack of significant changes in anthropometric characteristics and cardiovascular function.

Physical fitness decreased with age [36]. Moreover, as cardiovascular and respiratory system functions and immunity weaken with age, the risk of exposure to various diseases increases. Aerobic and resistance exercises improve physical fitness, can enhance the therapies for chronic diseases, reduce the risk of falls, and promote independent living through the development of bone density, muscle strength, and proprioception [9,23,37,38]. Lacroix’s study [39] conducted a balance and strength training program for healthy older people, dividing them into groups with and without exercise supervision. Both groups showed improved lower-extremity muscle strength and balance ability, whereas the supervised group had a more significant improvement. Therefore, the implication is that supervised exercise is more effective than exercising alone in improving physical strength and reducing the risk of falls. According to Al-Shreef’s study [40], aerobic exercise and resistance exercise three times a week for 24 weeks increased bone density and lowered the fracture risk in patients with type 2 diabetes, along with a significant increase in serum calcium and handgrip strength. The implication is that ongoing aerobic exercise and resistance training can help improve muscle strength and bone density. In addition, the study analyzed the effect of home workouts on muscular strength, endurance, and balance among healthy older people in the current era when physical activity is restricted due to the pandemic. Muscle strength, endurance, and balance ability improved when the exercise routine lasted for at least three weeks [41]. Our study results align with previous studies that show that all physical fitness variables significantly improve after continuous exercise. In comparing the improvement across the sexes, there was a relatively significant difference between males and females.

Studies have shown that exercise improves blood flow and increases the elasticity of blood vessels, which positively affects individuals with dyslipidemia and helps improve lipid profiles [42,43]. This study found significant differences in lipid profiles in TGs, LDL, and TC. This can also be attributed to drinking and smoking habits. Of course, some studies show that exercise does not significantly affect the improvement of lipid profiles and glucose [44,45,46]. Nevertheless, much research indicates that exercise has positive effects, such as lower cardiovascular disease prevalence, lipid improvement, and insulin sensitivity [8,37,38,47]. In addition, exercise has been shown to partially reverse the age-related physiological decline and improve individual work performance [48].

However, this study has the following limitations. First, since this study was limited to adults visiting a public health center in the Chungbuk area, the generalization of the results is limited. Second, it was impossible to recruit a control group due to the pandemic, which may be a weakness in the research experiment. Third, in this non-face-to-face study, we determined the authenticity of the exercise participation by participants recording their exercise type, intensity, time, and RPE. However, there were no direct visual means to confirm the participants’ exercise participation. Lastly, this study did not consider the effect of age, and the age range of the participants was wide. A study design that considers these limitations should be deployed in a follow-up study.

## 5. Conclusions

We found that the 24 weeks of non-face-to-face home exercise did not have a significant positive effect on body composition or cardiovascular function, but did have positive effects on physical fitness variables and caused improvements in lipid profiles. This suggests that this program would be suitable for improving physical fitness and dyslipidemia. To improve these results, we need to identify the presence of other underlying diseases in advance, ask participants to stop drinking and smoking, and add dietary therapy. Further, in a follow-up study, the above-mentioned changes should be supplemented by developing inducements for continuous exercise participation. In today’s world, people want to live mentally and physically healthy and happy lives. With the advent of customization in the digital age, more accessible and systematic non-face-to-face home workout systems using ICT should be established. This can contribute to promoting the health and welfare of citizens, as well as stimulate national economic growth.

## Figures and Tables

**Table 1:** Exercise program.

Classification	Exercise Program	%Heart Rate Max (Rating of Perceived Exertion)			Time
		Early	Middle	Later	
		1–8 Weeks	9–16 Weeks	17–24 Weeks	
Warm-up	Stretching Dynamic exercise (light)	20–40% (9–11)	20–40% (9–11)	20–40% (9–11)	5–10 min
Aerobic exercise	Walking (treadmill) Cycling	60–70% (13–15)	60–70% (13–15)	60–70% (13–15)	30–40 min
Strength exercises	Upper limbs (abdominals, arms, chest, and shoulders)	15–20 RM × 4 sets (13–15)	15–20 RM × 4 sets (13–15)	15–20 RM × 4 sets (13–15)	20–30 min
	Lower limbs (gastrocnemius and soleus, hamstrings, and quadriceps)				
Cool-down	Stretching	20–40% (9–11)	20–40% (9–11)	20–40% (9–11)	5–10 min
RM: repetition maximum					

**Table 2:** General characteristics of the participants.

Variables		Total (n = 58)	Male (n = 15)	Female (n = 43)	*t*	*p*
Age (years)		47.34 ± 11.04	49.53 ± 11.10	46.58 ± 11.05	0.890	0.377
Anthropometric characteristics	Height (cm)	161.46 ± 7.06	169.34 ± 6.64	158.71 ± 4.80	6.657	<0.001
	Weight (kg)	67.12 ± 13.23	76.55 ± 13.30	63.83 ± 11.65	3.511	0.001
	Body mass index (kg/m²)	25.59 ± 3.65	26.57 ± 3.37	25.24 ± 3.71	1.221	0.227
	Body fat (%)	33.23 ± 7.39	25.71 ± 6.80	35.85 ± 5.61	−5.702	<0.001
	Waist-hip ratio	0.91 ± 0.06	0.92 ± 0.08	0.90 ± 0.05	0.992	0.325
	Waist circumference (cm)	88.17 ± 11.03	92.26 ± 12.07	86.75 ± 10.42	1.694	0.096
Cardiovascular function	Systolic blood pressure (mmHg)	122.93 ± 10.73	130.20 ± 9.55	120.40 ± 10.02	3.301	0.002
	Diastolic blood pressure (mmHg)	81.21 ± 7.67	83.80 ± 9.07	80.30 ± 7.02	1.538	0.130
	Resting heart rate (bpm)	75.43 ± 9.83	74.67 ± 9.90	75.70 ± 9.91	−0.347	0.730
Physical fitness	Handgrip strength (kg)	29.56 ± 9.54	41.08 ± 10.75	25.55 ± 4.60	5.424	<0.001
	Sitting-rising test (points)	7.81 ± 1.71	7.20 ± 1.69	8.02 ± 1.69	−1.627	0.109
	Single-leg balance (seconds)	12.39 ± 9.91	12.10 ± 10.80	12.50 ± 9.71	−0.133	0.895
Blood profiles	Glucose (mg/dL)	97.16 ± 7.81	100.33 ± 7.36	96.05 ± 7.73	1.871	0.067
	Triglycerides (mg/dL)	99.62 ± 48.01	124.20 ± 56.72	91.05 ± 42.00	2.397	0.020
	High-density lipoprotein (mg/dL)	53.91 ± 16.98	45.20 ± 15.66	56.95 ± 16.52	−2.403	0.020
	Low-density lipoprotein (mg/dL)	113.20 ± 34.14	125.83 ± 39.64	108.70 ± 31.25	1.696	0.096
	Total cholesterol (mg/dL)	187.60 ± 33.44	196.20 ± 41.40	184.52 ± 30.08	1.002	0.329

Data are expressed as mean ± standard deviation, tested via an independent *t*-test between males and females.

**Table 3:** Changes in anthropometric characteristic variables after 24 weeks of non-face-to-face exercise.

Variables		Pre-Program (Mean ± SD)	Post-Program (Mean ± SD)	*t*	*p*
Weight (kg)	Total (n = 58)	67.12 ± 13.23	66.44 ± 12.51	1.506	0.138
	Male (n = 15)	76.55 ± 13.30	75.03 ± 11.26	1.368	0.193
	Female (n = 43)	63.83 ± 11.65	63.44 ± 11.60	0.820	0.417
Body mass index (kg/m²)	Total (n = 58)	25.59 ± 3.65	25.36 ± 3.59	1.371	0.176
	Male (n = 15)	26.57 ± 3.37	26.08 ± 2.87	1.306	0.213
	Female (n = 43)	25.24 ± 3.71	25.11 ± 3.81	0.747	0.459
Body fat (%)	Total (n = 58)	33.23 ± 7.39	32.77 ± 7.87	1.087	0.281
	Male (n = 15)	25.71 ± 6.80	25.08 ± 7.51	0.689	0.502
	Female (n = 43)	35.85 ± 5.61	35.46 ± 6.06	0.834	0.409
Waist-hip ratio	Total (n = 58)	0.91 ± 0.06	0.91 ± 0.06	−0.860	0.393
	Male (n = 15)	0.92 ± 0.08	0.92 ± 0.08	0.240	0.814
	Female (n = 43)	0.90 ± 0.05	0.91 ± 0.06	−1.458	0.152
Waist circumference (cm)	Total (n = 58)	88.17 ± 11.03	88.11 ± 11.09	0.099	0.921
	Male (n = 15)	92.26 ± 12.07	91.20 ± 11.35	0.704	0.493
	Female (n = 43)	86.75 ± 10.42	87.05 ± 10.93	−0.509	0.614

Tested via paired *t*-test.

**Table 4:** Changes in cardiovascular function variables after 24 weeks of non-face-to-face exercise.

Variables		Pre-Program (Mean ± SD)	Post-Program (Mean ± SD)	*T*	*p*
Systolic blood pressure (mmHg)	Total (n = 58)	122.93 ± 10.73	121.41 ± 9.12	1.245	0.218
	Male (n = 15)	130.20 ± 9.55	129.13 ± 8.29	0.516	0.614
	Female (n = 43)	120.40 ± 10.02	118.72 ± 7.83	1.124	0.267
Diastolic blood pressure (mmHg)	Total (n = 58)	81.21 ± 7.67	81.09 ± 7.93	0.159	0.874
	Male (n = 15)	83.80 ± 9.07	84.47 ± 10.00	−0.461	0.652
	Female (n = 43)	80.30 ± 7.02	79.91 ± 6.82	0.441	0.661
Resting heart rate (bpm)	Total (n = 58)	75.43 ± 9.83	74.28 ± 10.37	0.879	0.383
	Male (n = 15)	74.67 ± 9.90	71.53 ± 10.71	1.358	0.196
	Female (n = 43)	75.70 ± 9.91	75.23 ± 10.20	0.294	0.770

Tested via paired *t*-test.

**Table 5:** Changes in physical fitness variables after 24 weeks of non-face-to-face exercise.

Variables		Pre-Program (Mean ± SD)	Post-Program (Mean ± SD)	*t*	*p*
Handgrip strength (kg)	Total (n = 58)	29.56 ± 9.54	30.38 ± 9.48	−3.198	0.002
	Male (n = 15)	41.08 ± 10.75	42.49 ± 9,33	−2.876	0.012
	Female (n = 43)	25.55 ± 4.60	26.16 ± 4.72	−2.061	0.045
Sitting-rising test (points)	Total (n = 58)	7.81 ± 1.71	9.05 ± 1.13	−9.244	<0.001
	Male (n = 15)	7.20 ± 1.69	8.73 ± 1.22	−8.262	<0.001
	Female (n = 43)	8.02 ± 1.69	9.16 ± 1.09	−6.804	<0.001
Single-leg balance (seconds)	Total (n = 58)	12.39 ± 9.91	14.72 ± 11.82	−2.166	0.035
	Male (n = 15)	12.10 ± 10.80	17.97 ± 13.69	−3.498	0.004
	Female (n = 43)	12.50 ± 9.71	13.59 ± 11.05	−0.853	0.399

Tested via paired *t*-test.

**Table 6:** Changes in blood profile variables after 24 weeks of non-face-to-face exercise.

Variables		Pre-Program (Mean ± SD)	Post-Program (Mean ± SD)	*t*	*p*
Glucose (mg/dL)	Total (n = 58)	97.16 ± 7.81	98.02 ± 10.25	−0.635	0.528
	Male (n = 15)	100.33 ± 7.36	103.87 ± 10.85	−1.253	0.231
	Female (n = 43)	96.05 ± 7.73	95.98 ± 9.32	0.045	0.964
Triglycerides (mg/dL)	Total (n = 58)	99.62 ± 48.01	74.36 ± 48.56	4.299	<0.001
	Male (n = 15)	124.20 ± 56.72	98.13 ± 76.76	1.908	0.077
	Female (n = 43)	91.05 ± 42.00	66.07 ± 31.05	3.877	<0.001
High-density lipoprotein (mg/dL)	Total (n = 58)	53.91 ± 16.98	49.14 ± 16.94	3.203	0.002
	Male (n = 15)	45.20 ± 15.66	41.00 ± 14.30	1.313	0.210
	Female (n = 43)	56.95 ± 16.52	51.98 ± 17.01	2.934	0.005
Low-density lipoprotein (mg/dL)	Total (n = 58)	113.20 ± 34.14	102.29 ± 37.28	2.069	0.043
	Male (n = 15)	125.83 ± 39.64	109.47 ± 43.48	1.441	0.172
	Female (n = 43)	108.70 ± 31.25	99.73 ± 35.03	1.505	0.140
Total cholesterol (mg/dL)	Total (n = 58)	187.60 ± 33.44	167.14 ± 38.34	3.625	0.001
	Male (n = 15)	196.20 ± 41.40	170.93 ± 47.23	2.338	0.035
	Female (n = 43)	184.52 ± 30.08	165.79 ± 35.19	2.808	0.008

Tested via paired *t*-test.

## Data Availability

The data presented in this study are available upon request from the authors. Some variables were restricted to preserve the anonymity of study participants.
